# Miscellaneous Gleanings from Exchanges

**Published:** 1874-01

**Authors:** 


### Miscellaneous Gleanings from Exchanges.

### *MULTUM IN PARVO.*

Risks Attending the use of Bromide of Potassium.—Dr. James Norton, in *Glasgow Med. Journal* (Feb., 1873), details several cases, in which the use of bromide of potassium was followed by very unpleasant results. Case first was a woman aged forty, of strumous family. She was suffering from slowly progressive paralysis of one arm and leg of the same side. A mixture of bromide of potassium and ergot was given her. She became rapidly worse and died in a few days. The train of circumstances were such as to render it evident that the medicine hastened the result. Case second was a lady aged fifty. She complained of general muscular rheumatism. Bromide of potassium, in ten grain doses, three times per day, was prescribed for her. After using the medicine a few days she became gradually so confused and stupid in look, in fact, bloated in aspect, so unsteady in gait, and unable to walk, that she fell in getting out of bed. The medicine was discontinued, and these alarming symptoms speedily disappeared under the use of a bitter tonic. Case third was almost entirely similar, excepting that the medicine was given for pains in the legs and feet.—*Detroit Review of Medicine.*

Treatment of Inflamed Hæmorrhoids in Parturient Women.—A small piece of ice is to be placed in a little bag of rubber or gold-beater's skin, and applied to the tumor. The ice is to be replaced as fast as it melts. It is very raıely that at the end of an hour or two the pain is not very much diminished. The treatment may be continued for a part of the day and repeated the next, but care should be taken to envelop the bag with fine moist linen, so as to render the application less direct. It is also necessary, when about to discontinue the refrigeration, to let the ice melt completely, and permit the water to attain the temperature of the bed, to prevent a reaction which might reinduce pain.—*Gazette de Joulin* and *La France Mèdicale,* September, 1873.

Local Employment of Chlorate of Potash in Cancerous Sores.—In the *Berl. Klin. Wochenschrift*, Nov. 6, 1873, Dr. Burrow, of Konigsberg, advocates the local employment of chlorate of potash in the treatment of cancerous sores. His proceeding consists in sprinkling the sore with chlorate of potash in powder or crystals, and covering the whole with a wet compress. As the crystals of chlorate of potash exert a more powerful action than the powder, and excite greater pain, Dr. Burrow uses the powder first, and replaces it by the crystals when sensibility has been abated. One of the cases was a cancerous sore of the left arm, which healed completely after eight weeks' treatment. Three other cases were cancerous sores of the breast; one was lost sight of, the other two are under treatment, and healing well. The fifth case recorded was connected with a cancer which originated in the periosteum of the upper jaw and left cheek-bone, and then became ulcerated; in this case healing was complete in three months.—*Nashville Journal of Medieine and Surgery*, July, 1873, and *Journal of Materia Medica*, August, 1873.

Night Sweaes of Phthisis.—For the relief of this exceedingly troublesome symptom in this disease, a compaoatively new remedy is being used somewhat. It is the sulphate of atropia. Some of the patients are taking one-sixtieth of a grain in solution *ter in die;* some are taking one-hundredth of a grain at bed-time, and all are giving very good results. The success in this direction has already been sufficient to entitle it to a trial at least. A very excellent plan of managing these night sweats consists in sponging the patient in hot water, as hot as they can comfortably bear. If a patient is found sweating profusely in the night, take him out of bed, sponge him thoroughly with hot water, wipe him dry, replace his flannels, and put him back to bed. Sometimes a single sponging will arrest the sweating for two or three days.—*New York Medical Record.*

Amaurosis cured by the Extraction of Fangs.—Miss M., who had resided some time in the neighborhood of Nordhausen, had, in consequence of diminished power of vision in the left eye, consulted a physician, and had been under treatment for some time, but, instead of improving, had entirely lost the sight of the eye, when she consulted me on account of some trouble with her teeth. I extracted first the fang of the left canine tooth which had an abscess at its apex, and afterwards eight other fangs from the upper jaw. The next morning she came joyfully to me with the exclamation, "I can see again." I found that the pupil had regained its normal condition, and that the power of vision was re-established.—*Dental Cosma.*

VALERIAN IN DIABETES.—Dr. Bouchard, of *La Charité* Hospital, has been making a trial of valerian in diabetes. In diabetes without sugar the medicine did not seem to diminish the quantity of urine, but azoturia was obviously amended. The quantity of urea discharged in the twenty-four hours was much diminished; it decreased from forty-five grammes (about eleven drachms) to ten grammes (two drachms and a half.) The same results were observed in diabetes mellitus. In these cases, when there existed azoturia together with glycosuria, the quantity of urea always diminished under the influence of valerian. In some cases there was less excretion of water and sugar; but these effects seemed uncertain. Decrease in the production of urea was invariable. Valerian, therefore, prevents denutrition, and may be considered a saving remedy (médicament d'épargne).

Dr. Bouchard, in respect to this latter quality, quotes the customs of various Indian tribes, among whom the warriors during a month previous to going out to fight make use of valerian in every shape—in baths, in frictions, and internally. They ascribe to the substance the strength and courage which they feel in going through long marches, fatigue, and privation of food. This property of valerian has been observed in arsenic and bromide of potassium. Dr. Bouchard commenced with weak doses, which he gradually increased to one ounce of the extract, without having noticed any inconvenience.

TREATMENT OF CANCRUM ORIS.—Of all the local remedies or applications I have resorted to in such cases, I have never found any applicasion so useful or so effectual as hydrochloric acid. Neither nitric acid, nitrate of silver, nor chlorate of potash, nor any other remedy that I have ever tried, except hydrochloric acid, did I ever find of the least use to check cancrum oris. I have almost never found hydrochloric acid to fail to check the progress of this dreadful disease at once, and bring on a mrst rapid and healthy action in the part. Nor does it cause so much pain or suffering to the little patient as one would suppose, seeing that the gangrenous spot is almost entirely without feeling at this time. This acid is easily applied to the ulcer by means of a feather or small camel hair brush. I have cured many cases of cancrum oris by this means.—*Dr. McGreevy in British Medical Journal.*

BELLADONNA AS A PROPYLACTIC IN SCARLATINA.—When used, we prefer the formula of Flint, viz: "Dissolve one to three grains of ext. belladonna in an ounce of cinnamon water, adding a few drops of alcohol to prevent fermentation. Of this solution may be given two or three times a day, one drop for each year of the child's age, to be administered for two weeks, or longer if the danger should continue."—*Western Lancet.*

Syphilitic Onychia Treated with Acid Nitrate of Mercury.—Prof. G. Di Lorenzo finds that this painful affection, though it does not yield to the ordinary anti-syphilitic treatment, and often persists after other venereal symptoms have disappeared, is easily subdued by cauterizing with the acid mentioned above. He thinks his success may be due to a mercurial action of the remedy, but that, on the other hand, it is possibly attributable to his method of applying the caustic. He uses a small camel's-hair pencil, and moistens only the tip of it in the acid, remoistening it several times during the operation rather than to take up much acid at once. In this way he limits the caustic action to the bottom of the sulcus at the root of the nail, and avoids unnecessary destruction or inflammation of the adjacent tissues. He anæsthetizes the finger previously, and applies cold compresses afterward. The pain soon passes away. A tenacious eschar is left which excludes the air, and, when this falls off, the matrix of the nail is healthy, and capable of producing a perfect nail.—*Giornale Italiano delle Malattie Veneree e delle Pelle.*

Delirium Tremens at Rosevet Hospital.—The standard prescription for this condition is: ℞. Chloral hydrat, grs. xxx; potass. bromid, grs. lx. Mix. To be given at bed-time, and continued through the day in smaller doses if necessary. This is the treatment for a case of fully developed attack. Milder cases are treated by two drachms of the tincture of lupuline and a bottle of ale at bed-time, and in a mojority of instance this is all that is required; for as soon as the patient has secured a few hours of sleep the crisis is passed, and he will then begin to take his beef tea and milk, and come up rapidly.—*Medical Record.*

Treatment of Consumption at the Brompton Hospital.—T. G. Roddick, M.D., House Surgeon to Montreal General Hospital, writing from London to the *Canada Medical and Surgical Journal,* says that two thousand gallons of cod-liver oil are consumed in that institution in a year. Lead, turpentine and gallic acid are the sheet anchors there in hæmoptysis. Although the physicians are beginning to use ergotine hypodermically with moderate success, they do not depend on the latter remedy in severe cases.—*Medical Record.*

Removal of Ink Spots.—When these are of long standing it is difficult to get them out, since the iron has become thoroughly peroxidized and must be reduced. The following is recommended: Water, ½ litre; Hydrochloric acid, 100 grms; Tin salt, 100 grms. Moisten the spot with this solution, keeping it moist until the color disappears, and rinse with water.—*American Chemist.*

Treatment of Diabetes by arsenic.—Dr. Devergie (*La Frace Médicale,* March 19th,) observes that case of diabetes differ extremely and cannot all be treated alike. The cutting off of starchy diet is excellent, but it is painful treatment in many cases, and many patients are apt to relax much in attention to its exigencies. The privation of bread is kept to with great difficulty. He affirms that arsenical preparations have the power in many instances of getting rid of sugar from the urine. Called to treat a case of prurigo vulvæ, in a lady, Dr. Devergie prescribed arsenic, and it was soon discovered that the lady had glycosuria in addition to prurigo. This lady was cured of both maladies by means of the employment of arsenic. He then had the idea of treating all his diabetic patients with arsenic, and found the sugar often disappear completely, or become greatly lessened in quantity, without the patients much altering their diet.—*The Doctor.*

A Useful Soap.—The following is commended, by those who have tried it, for scrubbing and cleansing painted floors, washing dishes, and other household purposes: Take two pounds of white olive soap and shave it in thin slices; add two ounces of borax and two quarts of cold water; stir all together in a stone or earthen jar, and let it set upon the back of the stove until the mass is dissolved. A very little heat is required, as the liquid need not simmer. When thoroughly mixed and cooled, it becomes of the consistence of a thick jelly, and a piece the size of a cubic inch will make a lather for a gallon of water.—*Boston Journal of Chemistry.*

Rest in the Treatment of Consumption of the Lungs.—Dr. I. B. Berckert recommends the maintenance of rest of the thoracic walls as an important element of diseases of the lungs, for the same reason that it is resorted to in inflammation of joints and other organs. It is to this fact that he attributes the relief experienced by persons who apply plasters of different sorts to the walls of the chest in order to prevent or relieve the pain accompanying pulmonary troubles. In his own practice he depends on the application of strips of adhesive plaster.—*The Lancet,* Oct. 18, 1873.

Anti-Neuralgic Snuff.—The *Rivista Clinica di Bologna* mentions an anti-neuralgic snuff prescribed with success in cases of facial neuralgia, by Dr. Scriffignana. The base of the snuff is quinine, and its composition as follows: Citrate of quinine, ten grains; very strong exciting snuff (tobacco), fifteen grains. The medicament is said to act almost directly on the diseased nerve through the ethmoidal thread of the nasal ramus of Willis's ophthalmic, a branch of the fifth pair.—*London Lancet.*—*Dental Cosmos.*

Ether vs. Chloroform.—In an article in the *Brit. Med. Jour.*, March 8th, Dr. Hutchinson, of London, remarks, in reference to the general question as to the choice of anæsthetics, that we ought, with the exception of a few cases, to allow ether to supersede chloroform. At the same time he prefers chloroform to ether in the aged and the very young. Ether produces more cerebral excitement than chloroform; patients struggle more violently, and sometimes become unmanageable as if drunk; less air is allowed, and consequently there is greater liability to venous congestion about the head; all of which are attended with some degree of risk in a senile and degenerate brain. In young infants, his experience is that chloroform is exceptionally safe, and infinitely more convenient than ether in such operations as hare-lip, for instance, and he therefore gives it the preference.—*Canada Lancet, June*, 7.

Good Effects of Aconite in Acute Pneumonia.—Among other cases of interest which came under the observation of Dr. Murchison, in the male wards of St. Thomas's Hospital, London, was that of a boy aged fifteen, who was admitted with acute pleuro-pneumonia of the right side, and herpes labialis, and considerable increase of temperature. On the administration of eight minim doses of tincture of aconite, with liquor ammoniæ acetatis every four hours, the temperature at once came down, and the disease did not increase.—*British Medical Journal.*

To Keep Gum Arabic rrom Moulding.—Solutions of gum arabic soon mould and sour, and finally lose their adhesive property. It is said that sulphate of quinine will prevent this, while it imparts no bad odor of its own. The addition of a solution of a few crystals of this salt to gum arabic will prevent the formation of mould quite as effectually as carbolic acid, and by analogy it is safe to suppose that the same salt could be used in writing-ink, mucilage, and possibly glue.—*Druggists' Circular.*

Treatment of Chronic Eczema oe the Genitals.—Dr. DeMontmya recommends the use of the tincture of iodine in the treatment of the chronic eczema, and intertrigo of the genitals, more especially combined with the use of a lotion of one part of corrosive sublimate to 250 of water, a few drops of spirit being used to dissolve the corrosive sublimate.—*Medical Brief.*

Cerebro-Spinal Fever or Tetanus.—The following combination has been found very serviceable in both the above diseases: ℞. Potass bromidi, ʒv.; ext. ergot, fld., ℥j.; tr. calabar bean, ʒiij.; tr. opii, ℥ss.; aqua, ad., ℥viij.—M. Sig.—A tablespoonful every four hours.—*Canada Lancet.*

Treatment of Obstinate Forms of Epilepsy.—Dr. McLane Hamilton in the *Medical Record* says that the three drugs which give the best results in the treatment of this disease are the bromide of sodium and potassium, ergot and belladonna. He gives the Bromide in twenty grain doses thrice daily, and if necessary, in the course of a week in doses of thirty and even forty grains, until the toxic effect is produced. He considers the bromides more suitable to cases in which the attacks come on in the day time than in nocturnal epilepsy—belladonna and ergot are indicated in the *petit mal.* The hypodermic injection of the alkaloids seems to be the most efficacious mode of administration.—*The Canada Lancet.*

Treatment of Narcosis of Chloroform (*Bulletin Generale Therapeutique,* April 15th, 1873).—According to Dr. Baillēe, there is no more energetic means of overcoming the narcosis produced by chloroform than the introduction of a small piece of ice into the rectum. It can be pushed through the sphincter without the employment of much force. It immediately melts, producing a deep inspiration, which is the precursor of natural respiration and the re-establishment of cardiac functions. He recommends the same plan to be pursued in cases of apparent death in new-born infants.—*Phila. Med. Times.—Dental Cosma.*

Ergot in Dysentery.—M. Luton, of Rheims, *Gazette Heddomedal,* October, 1871, states that during an epidemic of dysentery the ergot of rye was prescribed successfully after the partial failure of other remedies generally used. The ergot appeared not only to act on the hemorrhagic element of dysentery, but the whole disease; and the mucous secretions, the griping colic and fever were equally relieved at the very commencement of the treatment.

On the Prevention of Pitting After Small-Pox.—Mr. Symes recommends as a local application to prevent pitting after small-pox, a mixture of one ounce of oxide of zinc to a pint of olive or linseed oil, which he has found most useful in a large number of cases. Mr. Symes has been in the habit also of keeping the room darkened.—*The Lancet.—Medical Brief.*

Dr. A. S. Hudson, in the *Pacific Medical and Surgical Journal* says: A mixture of pulv. steatite (soap-stone) two parts, and hyd. chlor. mitis one part, is the most elegant and effective dry application to the chafed skin of infants. Dry hyd. chlor. mitis, applied once or twice a day to tumid hemorrhoids situated about the anus, rarely fails to cure them in a few days.

